# Rare complications of anti-melanoma differentiation-associated gene 5 antibody-positive dermatomyositis: Time to nip them in the bud

**DOI:** 10.3389/fimmu.2022.1009546

**Published:** 2022-10-06

**Authors:** Jinming Yang, Bing Yan

**Affiliations:** Department of Rheumatology, West China Hospital, Sichuan University, Chengdu, China

**Keywords:** MDA5, dermatomyositis, pneumomediastinum, macrophage activation syndrome, spontaneous intramuscular hemorrhage

## Abstract

Anti-melanoma differentiation-associated gene 5 antibody-positive dermatomyositis (MDA5^+^ DM) is an infrequent autoimmune disease, which mainly distributes in Asians and females. MDA5^+^ DM usually presents various skin lesions and positive anti-MDA5 antibody (a myositis-specific autoantibody for itself) with amyopathic or hypomyopathic features. For MDA5^+^ DM patients, rapidly progressive interstitial lung disease is a common complication with a high-speed deterioration and a poor prognosis. Besides, there are other complications of MDA5^+^ DM patients, including pneumomediastinum, macrophage activation syndrome and spontaneous intramuscular hemorrhage. These complications were rare but lethal, so it is necessary to explore their diagnosis methods, therapies and potential mechanisms, which are helpful for early diagnoses and timely treatment. To date, several cases and studies have shown distinctive features, diagnoses and treatments of these three rare complications, and there are also some differences among them. In this review, we outlined the characteristics, administration and potential pathogenesis of these rare complications of MDA5^+^ DM.

## Introduction

Dermatomyositis (DM), one kind of idiopathic inflammatory myopathies (IIMs), is a heterogeneous autoimmune disease that manifests skin and muscle involvement usually complicating with the involvement and dysfunction of other organs. DMs can be divided into several subtypes according to their different myositis-specific autoantibodies (MSAs), among which anti-melanoma differentiation-associated gene 5 (anti-MDA5) antibody defines a distinct phenotype of DMs, namely anti-MDA5 antibody-positive DM (MDA5^+^ DM). MDA5^+^ DM is a relatively infrequent disease, which mainly prevails in Asian countries with a morbidity of 11-60%. Approximately 39-88% of MDA5^+^ DM patients are female (1).

MDA5, one member of the retinoic acid-inducible gene I (RIG-I)-like receptor families, acts as an intracellular RNA-sensor with its unique RNA-sensing mechanism to distinguish self and non-self RNAs. Upon infectious diseases, MDA5 selectively recognizes plus-strand RNAs from several viruses, which results in the inhibition of viral life cycle, the apoptosis of infected cells and the recruitment of inflammatory cells (2–4). For autoimmune diseases, the dysfunction of MDA5 may induce the development of autoimmune diseases, whereas the decreased expression of MDA5 reduces the onset of autoimmune diseases (5, 6). Anti-MDA5 antibody is not only a marker for the diagnosis of MDA5^+^ DM but also a useful predictor for disease activity and treatment response. The high titer of anti-MDA5 antibody is associated with acute phase or poor outcome while patients with low titer may have a relatively better prognosis (7, 8).

Clinically, MDA5^+^ DM is found to present skin lesions and positive MSA but has no muscle disorder or mild myopathic manifestation, so it also falls into the category of clinically amyopathic dermatomyositis (CADM) due to its amyopathic or hypomyopathic features (9). Identical to other types of DMs, the skin manifestations of MDA5^+^ DM include heliotrope rash with edema around eyes, facial erythema, a V-sign (for erythema on the anterior chest), a shawl sign (for erythema on the back and shoulders), Gottron’s papules (for zinzolin papules distributing on the dorsal part of elbow, knee, metacarpophalangeal joints or interphalangeal joints, covering with scale on the surface) and mechanic’s hands (for keratinized and cracked status of radialis palmar skin). The skin lesions can present alone or in combination. These typical skin lesions combined with positive anti-MDA5 antibody are reasonable for the diagnosis of MDA5^+^ DM.

It has been reported that rapidly progressive interstitial lung disease (RP-ILD) is one of the most common complications of MDA5^+^ DM. The patients present dyspnea with a high-speed deterioration and hypoxemia, with or without secondary pulmonary infections. Serving as a main assessment tool, pulmonary high-resolution computed tomography (HRCT) shows signs of consolidation and ground-glass opacity. The HRCT pattern of MDA5^+^ DM-related ILD is unclassifiable because its reticulation is inconsistent with typical HRCT patterns (including usual interstitial pneumonia, organizing pneumonia and nonspecific interstitial pneumonia) (10). Surgical lung biopsy in RP-ILD shows diffuse alveolar damage (DAD), presenting a pathological pattern of intra-alveolar membranous formation and diffuse alveolar collapse (11). Owing to rapid exacerbation of the complication and lack of effective therapies, MDA5^+^ DM patients are undertaking a poor prognosis (12).

**Figure 1:**
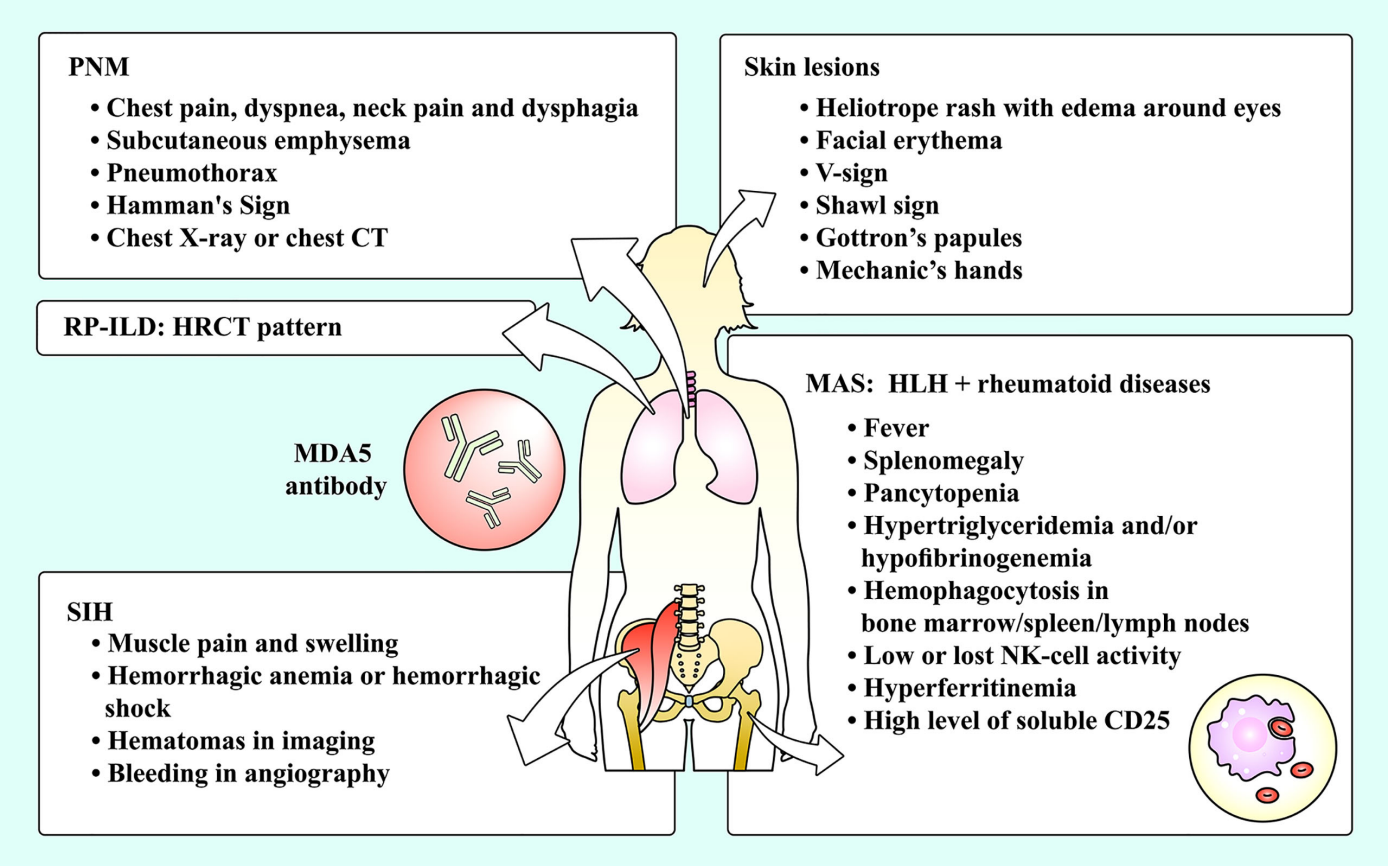
The complications of MDA5^+^ DM and their manifestations. Skin lesions and RP-ILD are common complications among MDA5^+^ DM patients, while PNM, SIH and MAS are relatively rare complications of MDA5^+^ DM patients. DM, dermatomyositis; HLH, hemophagocytic lymphohistiocytosis; HRCT, high-resolution computed tomography; MAS, macrophage activation syndrome; MDA5, melanoma differentiation-associated gene 5; NK, natural killer; PNM, pneumomediastinum; RP-ILD, rapidly progressive interstitial lung disease; SIH, spontaneous intramuscular hemorrhage.

MDA5^+^ DM patients also have other complications, such as pneumomediastinum (PNM), macrophage activation syndrome (MAS), spontaneous intramuscular hemorrhage (SIH), etc (**Figure 1**). These complications were previously reported to be rare but lethal. In this review, we outlined the characteristics, administration and potential pathogenesis of these rare complications of MDA5^+^ DM. This review had three purposes. Firstly, the rare complications may be easily ignored by clinicians, so actual incidence may be underestimated. But due to the lethality, we must be vigilant and explore the approaches for an early diagnosis. Secondly, there are few systematic guidelines providing suggestions for the treatment of the complications, which is the second reason for writing this review. Thirdly, the occurrence of complications may be closely related to the mechanisms of MDA5^+^ DM, so the exploration of complications may help us further understand its pathophysiological process.

## PNM and MDA5^+^ DM

PNM, also known as mediastinal emphysema and Hamman’s Syndrome, is a syndrome characterized by free air pathologically invading the mediastinum. PNM includes spontaneous PNM and secondary one. Differing from secondary PNM (usually due to trauma and iatrogenic factors), spontaneous PNM is contributable to the non-traumatic and ambiguous pathophysiological mechanisms. Currently, spontaneous PNM is reported to be associated with DM as well as severe asthma, pneumonectasis, etc. (13, 14) It was reported that the morbidity of spontaneous PNM in MDA5^+^ DM patients reaches more than 15% and the mortality elevated up to approximately 60% (15).

### Clinical manifestation and diagnosis

The symptoms of PNM in MDA5^+^ DM patients are similar to those of other causes. The patients may manifest chest pain, dyspnea, neck pain and dysphagia, often accompanied by subcutaneous emphysema. Sometimes it may complicate with pneumothorax (16). Upon cardiac auscultation, churning sounds synchronizing with the heartbeat can be heard, which was firstly reported by Louis Hamman and was addressed as Hamman’s Sign (17). It is considered the consequence of the repeated squeezing of air-filled mediastinum between the chest wall and heart. However, since these symptoms sometimes are unspecific, the patients with mild conditions possibly mistake them for other disorders and fail to see a doctor, or are misdiagnosed or undiagnosed by physicians. Therefore, the actual incidence may be underestimated.

For making a confirmed diagnosis, chest imaging can be useful. The utilization of plain chest X-ray can help to confirm the existence of air within the mediastinum but is sometimes restricted to a small amount of free air (14). If conditional, a chest CT scan may be necessary because the integration of plain chest X-ray and CT scan performs better with the accuracy reaching closely to 100% (14, 16). As for laboratory tests, there was no significant difference among MDA5^+^ DM patients with PNM compared with those without (18). However, of MDA5^+^ DM patients with PNM, non-survivors were predisposed to low alveolar-arterial oxygen difference (A-aDO_2_) and elevated aspartate transaminase (AST) and lactate dehydrogenase (LDH) in comparison with survivors, but not all of the liver enzymes and muscle enzymes might rise (15, 18). In conclusion, the diagnosis of PNM needs an evaluation procedure of adequate history, physical examination and chest imaging while it lacks distinction on laboratory tests.

### Risk factors

Among MDA5^+^ DM patients, PNM is usually accompanied by interstitial pulmonary changes, but their relationship remains controversial. A previous study found that DM patients suffering from PNM had a high prevalence of positive anti-MDA5 antibody and RP-ILD (19). Similarly, another study also showed that there were various degrees of pulmonary changes among MDA5^+^ DM patients with PNM. And the deaths in the PNM group also showed worse pulmonary changes (18). Owing to the high incidence of ILD, it is considered that PNM is closely linked to RP-ILD of MDA5^+^ DM, which reflects the severity of pulmonary involvement and suggests a poor prognosis. Nevertheless, a current case of MDA5^+^ DM reported that ILD got improvement after treatment, during which, however, PNM occurred (20). The paradoxical procedure between them two is inconstant with the opinion that PNM may be a presentation of the exacerbation of ILD. On the other hand, there is an opposite view that vasculopathy rather than ILD is a prognostic risk factor for PNM in DM patients. The vasculopathy commonly represents cutaneous lesions or ulcers among the patients. Ma and colleagues investigated the relationship between PNM and several potential risk factors (including cutaneous ulcer and ILD) and found that cutaneous ulcer was the only risk factor for DM patients with PNM (19). An earlier study reviewed several reported cases and found a high occurrence of skin ulcers or cutaneous vasculitis in DM patients with PNM, among whom some did not present ILD (21). In that study, a special case was reported. He was a DM patient complicated with ulcerative skin, oral ulcers and hemoptysis, which suggested systemic vasculopathy. Bronchoscopy showed non-infectious necrosis of bronchial wall. The findings indicated that bronchial wall necrosis might be induced by systemic vasculopathy and linked to the air leakage causing PNM (21). These studies supported the relationship between cutaneous ulcers and PNM. However, they investigated simply among DM and did not focus on the subtype MDA5^+^ DM. The study on MDA5^+^ DM found that there was no significant difference between individuals with PNM and those without, but the result might be limited to by the small number of samples (18). More animal and large population-based studies need to be conducted between PNM and RP-ILD or cutaneous vasculopathy among MDA5^+^ DM.

Despite of contentious relationship between PNM and other risk factors (ILD or vasculopathy), the death of MDA5^+^ DM patients may not only hinge on the outbreak of PNM. Yoshida et al (22) found that DM patients with PNM usually died of ILD-related respiratory failure although PNM indeed served as a fatal complication of the patients. The author viewed that PNM itself did not act as a factor for poor prognosis. In accordance, MDA5^+^ DM-related PNM patients with mild pulmonary interstitial changes had better survival than those with severe ones, which was reported by Yamaguchi et al. (18) Moreover, the speed of air leakage may also be a potential prognostic factor. “Slow leak”, a new definition by Zhang et al (23), was defined as dyspnea progressing for more than 3 days. The slow air leakage of PNM was found to be a predictor of better prognosis in DM patients. It seems to be relatively reasonable because slow air leakage means the slow and mild process of PNM. But there has not been a detailed epidemiological survey and prospective study about the cause of death among MDA5^+^ DM patients. Also, due to the complexity of co-existing complications, it is a challenge for physicians to evaluate the specific causality.

### Pathogenesis

The pathogenesis of the occurrence of spontaneous PNM in MDA5^+^ DM is not explicit. The basic reason is possibly the elevated pressure within the alveolar space. In virtue of animal experiment, Macklin et al. (24) deemed that elevated pressure in the alveoli caused the alveolar rupture, after which air spread to the pulmonary interstitium and diffused to the mediastinum and subcutaneous area, leading to PNM and subcutaneous emphysema. This was called as Macklin effect for spontaneous PNM. Therefore, the factors inducing alveolar hyperinflation may drive PNM, including fierce cough, asthma, pulmonary function test, ILD exacerbation, etc.

As for PNM arising among the MDA5^+^ DM patients, there are three possibilities. First, PNM may be associated with the MDA5^+^ DM-related ILD. ILD presents inflammatory cell infiltration and the distortion of lung architecture, which causes the fragility of the alveoli. If there are some factors aggravating the alveolar pressure, the alveoli are more likely to be destroyed and lead to PNM. Nevertheless, there has not been evidence of the high level of alveolar pressure and alveoli destruction. Indirectly, some current cases of coronavirus disease 2019 (COVID-19) reported that PNM was in connection with DAD (25–27), the latter usually occurring in COVID-19 as well as acute interstitial pneumonia (AIP), acute respiratory distress syndrome (ARDS) (28–30). Similarly, the histopathology of the MDA5^+^ DM-related ILD is also DAD (11). In light of the clues offered by COVID-19, it is hypothesized that PNM in MDA5^+^ DM is attributed to ILD-related DAD. Alternatively, as one of the potential influence factors, DM-related vasculopathy is probably another driver for PNM. MDA5^+^ DM is found to be strongly associated with type I interferon (IFN-I) signaling (31), a contributor to the injury of endothelium and the release of endothelin (32). The endothelin is a vasoconstrictor that causes focal ischemia (33). Meanwhile, the formation of thrombosis also may join in the ischemic process, because MDA5^+^ DM was reported to show upregulation of von Willebrand factor (vWF), a pro-coagulant (32). Therefore, the repeated injury and vasoconstriction of vasculatures mediated by IFN-I signaling occurs in the lung and alveoli, after which the alveolar tissue may tend to rupture due to ischemia. The case of PNM complicated by the necrosis of bronchial wall partly supported that continuous inflammation of vasculatures might cause damage to its surrounding structures (e.g., bronchial wall) (21). Third, PNM in MDA5^+^ DM may be the result of the use of glucocorticoids. PNM was observed during or after the treatment of glucocorticoids in most patients with connective tissue diseases (CTDs) including DM (22, 23). Theoretically, glucocorticoids may weaken the pulmonary interstitium by affecting protein metabolism. But a study found that intravenous methylprednisolone pulse therapy was not a predictor of the development of PNM in CTDs (34). But more study on PNM in MDA5^+^ DM is scarce.

### Treatment

At present, there has been no unified treatment for PNM in MDA5^+^ DM patients. PNM is inclined to develop along with the presentation of RP-ILD among MDA5^+^ DM patients. Multidisciplinary therapy with the combination of high-dose glucocorticoid, aggressive immunosuppressants (cyclophosphamide or tacrolimus), tofacitinib and rituximab with or without surgery is used as a pattern for the patients. Several cases reported that PNM patients with refractory ILD got remission after the use of combined immunosuppressive therapy (35–37). In discrepancy, Zhang et al. (23) found that all the treatments, including steroid pulse therapy, immunosuppressive agents, intravenous immune globulin (IVIG) and surgical drainage, were not associated with the survival of DM-related PNM patients. Accordantly, PNM survivors of MDA5^+^ DM were not significantly different from non-survivors in the aspect of glucocorticoid and immunosuppressive therapy (15). Notably, regardless of different survival status, most PNM patients in these observational studies received therapies, so the characteristic baseline was intrinsically balanced and an insignificant difference was expectable.

Some patients may show hypoxemia influenced by PNM and need oxygen supplement. However, noninvasive positive pressure ventilator (NPPV) was reported as a risk factor for the prognosis of PNM, because NPPV gives positive pressure, cause alveoli to burst and to deteriorate PNM, which is consistent with Macklin effect (15). A case of MDA5^+^ DM reported that PNM occurred after the use of NPPV, while the combination of drugs and oral intubation with mechanical ventilation improved PNM (35). It suggested that early mechanical ventilation may be a better alternative to improve the anoxic status of PNM. In addition, other approaches (cough suppression for example) which may reduce alveolar pressure are also recommended (**Figure 3**). More prospective intervention studies on the effectiveness of glucocorticoids and immunosuppressants for PNM are warranted.

There are also other choices. Another successful case found lung transplant might be useful for PNM in MDA5^+^ DM patients (38). An observational study by De Giacomi et al. (39) reported that no deaths of PNM in CTDs was observed after given the measures of observation only, oxygen therapy and chest tube drainage. Despite the effectiveness of surgical methods, they may be limited by the acceptance of patients, physical conditions and postoperative administration.

## MAS and MDA5^+^ DM

MAS is a form of hemophagocytic lymphohistiocytosis (HLH). HLH, also named hemophagocytic syndrome, is characterized by over-response of inflammation mediated by primary (familial) or secondary (acquired) immune dysregulation. When HLH is secondarily driven by autoimmune disorders or rheumatoid diseases, it is called MAS (40). MAS is considered a fiercely life-threatening complication for patients with rheumatoid diseases, with an evaluated mortality as high as 20% (41). In rheumatology, the most common etiology of MAS is systemic juvenile idiopathic arthritis (sJIA), systemic lupus erythematosus and adult-onset Still disease (42–44). Only a minority of published literatures reported MDA5^+^ DM complicated with MAS and showed a poor prognosis. By the means of ^18^F-fluorodeoxyglucose positron emission tomography/computed tomography (PET/CT) scan, a study showed that positive anti-MDA5 antibody was related to the development of MAS among IIM patients, indicating a significant correlation between MAS and MDA5^+^ DM (45).

### Clinical manifestation and diagnosis

The manifestations of HLH/MAS are a mixture showing simultaneously or in succession, including continuous fever, splenomegaly, hypertriglyceridemia, hypofibrinogenemia, pancytopenia, hyperferritinemia and hemophagocytosis in bone marrow. The elevation of soluble interleukin-2 (IL-2) receptor (also known as soluble CD25) and the decrease of natural killer (NK) cell activity may also be observed. Some critically ill patients with HLH/MAS may present multiorgan dysfunction, such as liver dysfunction, central nervous system disorders and renal failure (41).

MAS not only can be the first manifestation of MDA5^+^ DM, but also appears along with the progression of MDA5^+^ DM. Some published case reports revealed the relationship between MAS and MDA5^+^ DM (46–54). Thereinto, MAS sometimes acted as the initial presentation before the confirmation of MDA5^+^ DM in juvenile and adult populations (46, 47, 49). Conversely, sometimes MDA5^+^ DM might onset firstly and MAS developed successively (48–53). Among these cases of MDA5^+^ DM patients, most cases reported obviously high level of serum ferritin, among which some even reached more than 10,000 ng/ml (46–54). Elevation of transaminase and/or persistent fever also could be common features among some cases (46–48, 51–54). MDA5^+^ DM patients with HLH/MAS might be combined with pre-existing infections (48), but most cases of MDA5^+^ DM-related MAS were reported no evidence of pre-existing infections on admission. Even so, some patients might suffer from secondary or opportunistic infections at hospitalization (46–48), so it is still vital to exclude infections fully and repeatedly.

The diagnosis of MDA5^+^ DM-related MAS needs to satisfy the HLH-2004 criteria based on the condition of MDA5^+^ DM (55). MDA5^+^ DM alone cannot explain all the presentations. Therefore, MAS should also be discerned and identified when a MDA5^+^ DM patients develop the manifestations mentioned in the criteria. However, when a patient is suspected of developing MAS but does not fully meet the HLH-2004 criteria, we can also use some extra scoring tools. MAS/sJIA score and haemophagocytic syndrome diagnostic score (HScore) are available tools as a supplement and judgment (56, 57).

### Pathogenesis

MAS is a process characterized by over-release of cytokines and overactivation of macrophages and T lymphocytes (58, 59). Several studies showed that elevated circulating cytokines, including IL-­1, IL-­6, IL-­18, IFNs and tumor necrosis factor (TNF), were related to the onset of MAS and caused cytokine storm syndrome (59–61). Alternatively, activated macrophages in MAS was also closely associated with IFN-­γ (also namely type II interferon, IFN-II), which induces the production of a string of cytokines and is regulated by the cytokines in turn (60).

There are some “predisposed” immune backgrounds in which MDA5^+^ DM is susceptible to MAS. Gushing release of cytokines in MDA5^+^ DM is one of the backgrounds. Several studies found that a majority of MDA5^+^ DM or CADM patients presented elevated levels of serum cytokines, including IFN-α, IFN-β, IFN-γ, IL-1β, IL-6, IL-10, IL-18 and IL-12 (62–64). Hensgens et al. (65) found that signaling pathways of IFN, IL-1, IL-10 and IL-18 families upregulated in MDA5^+^ DM in comparison to healthy individuals. Increased C-C motif chemokine ligand 2 (CCL2), C-X-C motif chemokine ligand 9 (CXCL9) and CXCL10 could be also observed in CADM patients (64). What’s more, another background is autoantibodies (including anti-MDA5 antibody and non-anti-MDA5 antibodies) in MDA5^+^ DM, whose actions promote cytokine release. Relying on RNA binding, immune complexes formed by anti-MDA5 antibody and MDA5 could induce the activation of IFN-I (66). Recently, one study found that non-anti-MDA5 antibodies in MDA5^+^ DM could directly bring about the production of IFN-­γ from peripheral blood mononuclear cells (PBMCs) (67). When the patients are complicated by viral infection, viral RNA-sensing MDA5 can also promote the activation of IFN-I pathway (2).

**Figure 2:**
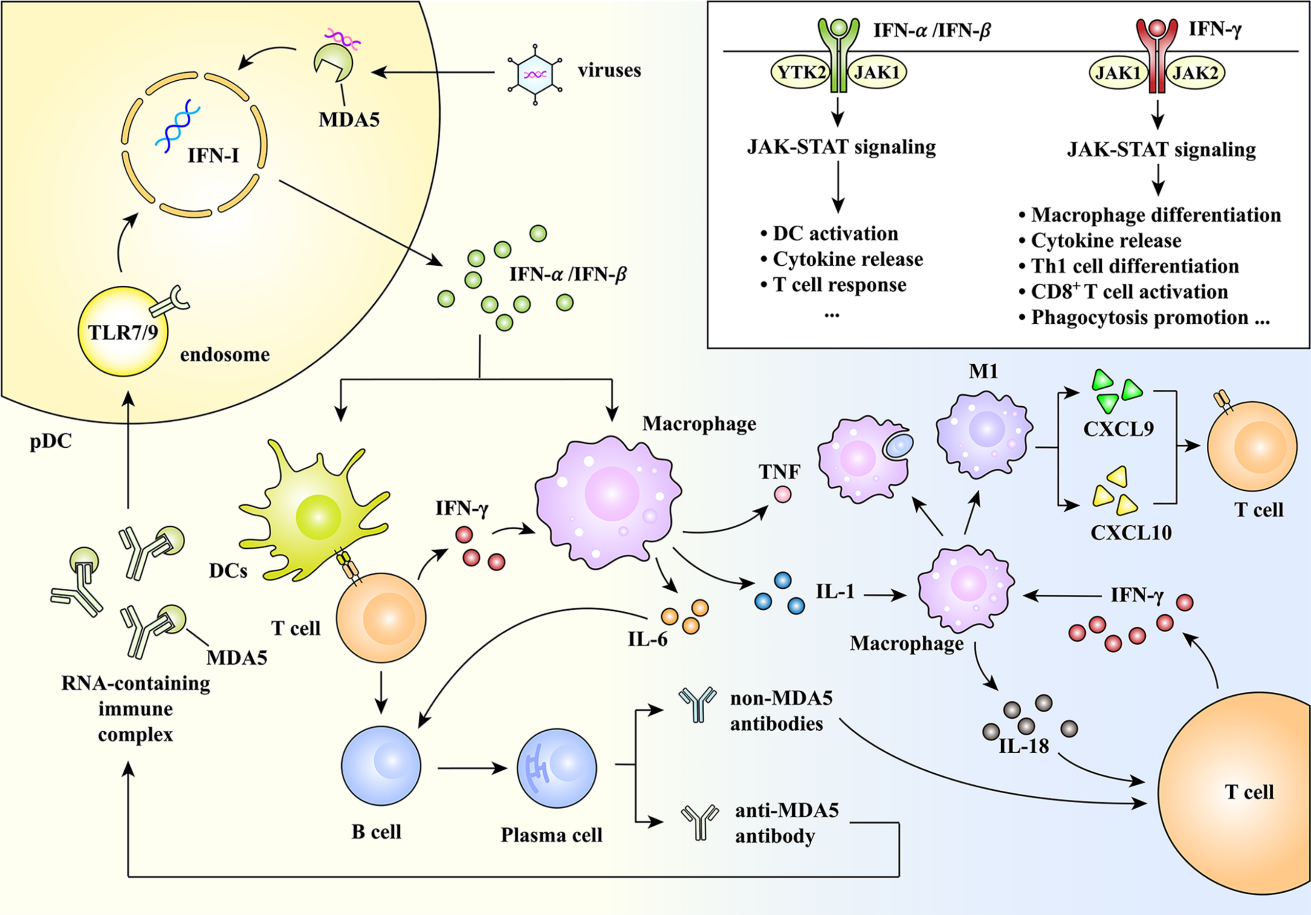
A brief introduction of the potential pathways of MDA5^+^ DM-related macrophage activation syndrome (MAS). In MDA5^+^ DM, some external and internal factors (e.g., virus infection) stimulate pDCs to release IFN-α or IFN-β through IFN-I signaling. On the one hand, IFN-α or IFN-β activates macrophages and induces the release of pro-inflammatory cytokines, including IL-6, IL-1 and TNF. IL-6 promotes B cell to differentiate to plasma cell, and IL-1 triggers the pro-inflammation of macrophages. Alternatively, IFN-α or IFN-β also stimulates dendritic cells to be mature, followed by the effective response of T cells and B cells. Plasma cells differentiated by B cells produce autoantibodies, including anti-MDA5 antibody and non-MDA5 antibodies. The RNA-binding immune complexes formed by anti-MDA5 antibody and MDA5 activate IFN-I in turn, while non-MDA5 antibodies directly induce the production of IFN­γ. This is a vicious cycle that promotes autoimmune inflammation. Also, the macrophages promoted by IL-1 release IL-18, which induces the synthesis of IFN­γ. IFN­γ can lead macrophages to get enhanced phagocytic function and to transform to pro-inflammation (M1) subtype. M1 subtype produces chemokines to recruit T cells to inflammatory sites. Thereinto, IFN-I and IFN-II signaling are both mediated by JAK-STAT pathways, thereby followed by different effects in the downstream. Together, these complex cellular interactions mediate the occurrence of MAS. CXCL, C-X-C motif chemokine ligand; DC, dendritic cell; IFN, interferon; IL, interleukin; JAK, Janus kinase; MAS, macrophage activation syndrome; MDA5, melanoma differentiation-associated gene 5; pDC, plasmacytoid dendritic cell; STAT, signal transducer and activator of transcription; Th1, T helper 1; TLR9, Toll­like receptor 9; TNF, tumor necrosis factor; YTK, tyrosine kinase.

MDA5^+^ DM-related MAS may be mediated by IFN-I and IFN-II siganlings, where JAK-STAT signaling acts in autoinflammatory and autoimmune process. First, in IFN-I signaling, IFN-α or IFN-β is released by plasmacytoid dendritic cells (pDCs), stimulates dendritic cells to be mature and activate macrophages. T cells and B cells start to mediate immune response and plasma cells produce anti-MDA5 antibody and non-anti-MDA5 antibodies. These autoantibodies mediate two IFN signalings and create a vicious cycle that promotes autoimmune inflammation. Successively, IFN-­γ is a mediator which functions the polarization of macrophages to pro-inflammatory phenotypes (M1) and intensifies the phagocytic ability of macrophages (68, 69). It is also an up-regulator on the expression of CXCL9 and CXCL10, which act in the process of Th1 (T helper 1) cell response (70). In conclusion, MDA5^+^ DM-related MAS may be the result of the over-released cytokines and the interactions of immune cells under the condition of the immune risk states (**Figure 2**).

### Treatment

Currently, there is no consensus on the treatment of MDA5^+^ DM complicating with HLH/MAS. Inhibiting HLH/MAS requires more aggressive anti-inflammatory and immunosuppressive therapies, which can also be assisted by plasma exchange and biologic agents.

MAS is attributable to overactive immune system, so halting the fierce immune dysfunction is one of the methods to control the disease. High-dose glucocorticoids and immunosuppressants are usually used firstly in the cases of MDA5^+^ DM-related MAS (46–48, 51, 52). At the stage of MAS, glucocorticoid was treated with 1000mg intravenously for about three days and tapered then (46, 48, 51, 52). As for immunosuppressants, cyclosporine was chosen in most cases (46, 50, 53, 54). A previous study found that cyclosporine was effective for HLH/MAS (71). Etoposide, a chemotherapeutic drug for ablating over-activated immune cells, is one of the drugs recommended by HLH-2004 criteria (55). Some studies found that etoposide played a role in HLH/MAS possibly by selectively eliminating pathologic T cells (72–74). In the case of MDA5^+^ DM-related MAS, etoposide was used and showed efficiency (46). In HLH-2004 protocol, glucocorticoid, cyclosporine and etoposide are recommended as the initial therapy at the week 1-8 in HLH/MAS (55).

Silencing cytokine signalings may be effective for MDA5^+^ DM with MAS. As is mentioned above, there are some inflammatory cytokines (e.g., IL-1 and IL-6) elevating in MDA5^+^ DM-related MAS. In inflammatory diseases, IL-1 receptor antagonist (IL-1RA) acts as a competitor against IL-1 and is competitively combined with IL-1 receptor type 1 (IL-1R1), blocking the pro-inflammatory effect of IL-1. Anakinra is one of IL-1RAs. For a patient with MDA5^+^ JDM, anakinra was used to control the disease successfully (46). Another case of MDA5^+^ DM also got partial diminishment of C-reaction protein and fever after the treatment of anakinra, suggesting the ease of inflammation state (75). Previous studies found that patients with HLH/MAS showed improvement after the use of anakinra (76, 77). Alternatively, blocking IL-6 may be also helpful. Tocilizumab is an IL-6 receptor antagonist that binds to soluble and membrane IL-­6 receptors. The case of MDA5^+^ DM-related MAS showed the success of Tocilizumab on MAS (50). Another MDA5^+^ DM cases with RP-ILD undergoing the treatment of Tocilizumab also showed decrease of ferritin and improvement of respiratory disorder, suggesting that Tocilizumab may be a salvage therapy for MDA5^+^ DM (78). Besides, we can also treat the patients by inhibiting the JAK-STAT signaling, which is related with the process of MDA5^+^ DM-related MAS. Tofacitinib, a JAK inhibitor, was reported to be effective in refractory MDA5^+^ DM-related ILD (79, 80). The published case showed that Tofacitinib on top of other immunosuppressants was effective for MDA5^+^ JDM-related MAS (46). Ruxolitinib is another JAK inhibitor. Although it has not been used to treat MDA5^+^ DM-related MAS, it was reported that ruxolitinib showed effectiveness for refractory HLH/MAS, which offers a new potential therapy for MDA5^+^ DM-related MAS (81–83).

We can also block MAS by inhibiting B cell response. Rituximab, an anti-CD20 monoclonal antibody, induces B cell apoptosis and decreases the production of autoantibodies. Several cases provided common evidence that rituximab showed its possible effectiveness on MDA5^+^ DM-related MAS (50, 51, 54), but there is not large study to investigate the efficacy among MDA5^+^ DM-related MAS.

Besides, high-dose IVIG is increasingly used for the treatment of autoimmune diseases owing to its role in anti-inflammation. The administration of IVIG in high concentration is considered to join in autoantibody neutralization, cytokine network interruption and cell proliferation regulation (84). Some MDA5^+^ DM-related MAS patients treated with IVIG and anti-inflammation therapy successfully got improved (46, 54).

Plasmapheresis is also considered a possibly effective remedy (85). Shirakashi and colleagues (85) found that through plasma exchange anti-MDA5-positive DM survivors showed decreased titer of MDA5^+^ DM compared with that before the therapy. Likewise, a few cases showed repeated plasma exchange reduced the titer of anti-MDA5 antibody, followed by the improvement of illness (86). A current real-world analysis found that plasma exchange combined with standard immunosuppressive therapy was also effective for rheumatic diseases-associated MAS, which was associated with the clearness of over-released cytokines (87).

Notably, during the use of high-dose glucocorticoids and immunosuppressants, some MDA5^+^ DM patients developed secondary infections, which might be associated with the immunosuppression status (46–48). Therefore, in addition to repeatedly searching for the evidence of infections, we should also commence early anti-infection therapy because infection is also an inducer of HLH/MAS.

**Figure 3:**
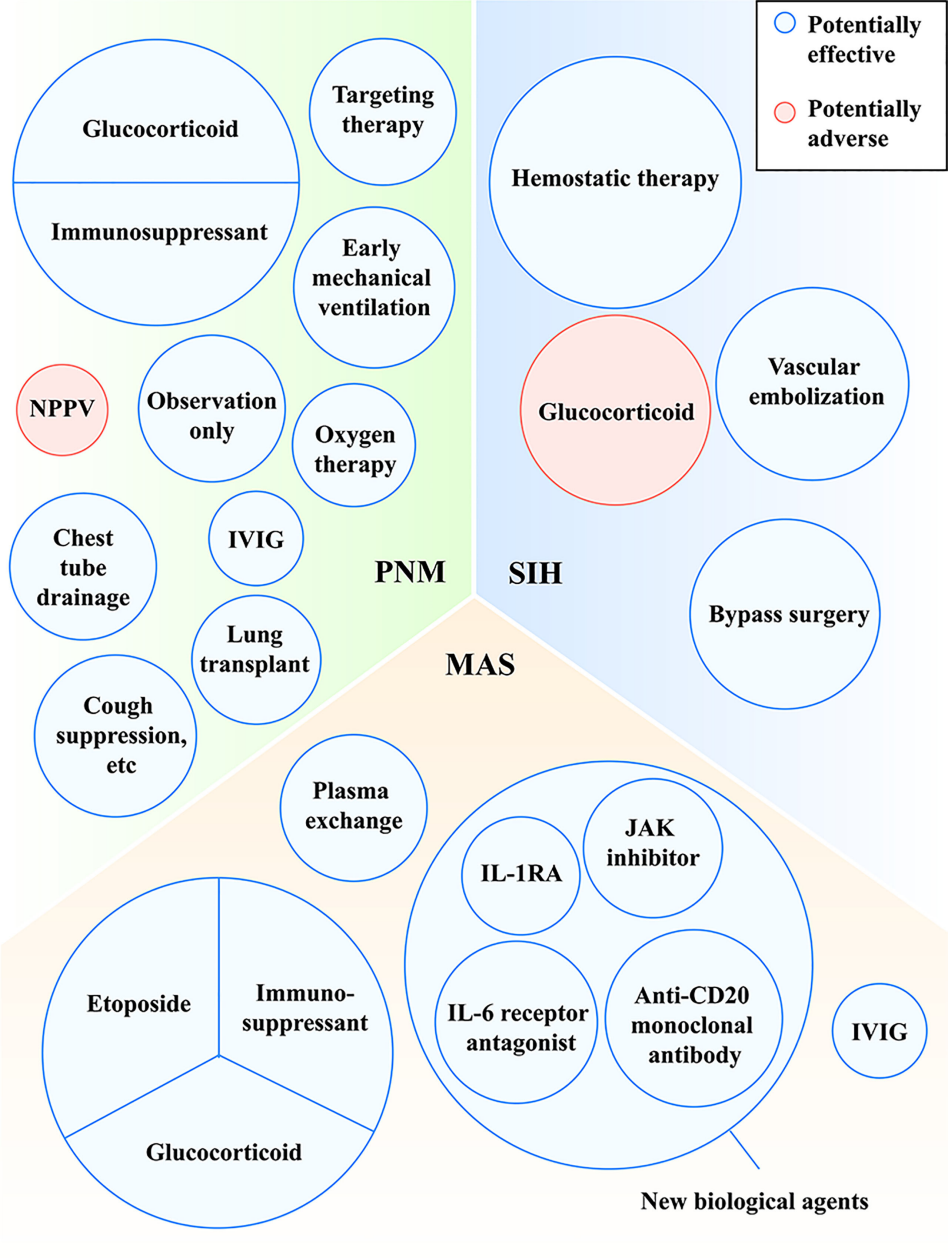
Treatments with potentially effective and potentially adverse effects in MDA5^+^ DM-related PNM, MAS and SIH. IL, interleukin; IL-1RA, interleukin-1 receptor antagonist; IVIG, intravenous immune globulin; JAK, Janus kinase; MAS, macrophage activation syndrome; MDA5, melanoma differentiation-associated gene 5; NPPV, noninvasive positive pressure ventilator; PNM, pneumomediastinum; SIH, spontaneous intramuscular hemorrhage.

Nevertheless, these cases did not achieve a unified treatment and provide the principle for MDA5^+^ DM with MAS. More studies are needed to unfold the treatment of MDA5^+^ DM-related MAS and more new drugs are needed to explore (**Figure 3**).

## SIH and MDA5^+^ DM

SIH is a rare but life-threatening complication of DMs. It presents short-term, insidious and non-traumatic muscular hemorrhage or hematoma, which may cause disseminated intravascular coagulation (DIC) or hemorrhagic shock. Sometimes it is also called hemorrhagic myositis. Currently, there were a minority of cases reporting the relationship between SIH and DMs, and MDA5^+^ DM complicated with SIH are much fewer. Even so, owing to its fatality, we cannot ignore it. And we should notice a possibility that its actual prevalence and mortality may be higher than those reported before.

### Clinical manifestations

Kono et al. (88) firstly reported a case of MDA5^+^ DM with SIH, who presented skin lesions and pulmonary manifestation with the absence of muscle involvement at the first time. However, he suffered repeated muscle pain and swelling of bilateral brachia in the duration of hospital stay, which showed hematomas in brachial muscles in magnetic resonance imaging (MRI) and bleeding of axillary artery in angiography. Vessel bypass surgery was performed as a solution. Currently, a published literature also reported a series of cases of MDA5^+^ DM with SIH in their cohort (89). In addition, there were also some non-MDA5^+^ DM patients with SIH, a majority of whom were positive for anti-Ro52 antibody (89–91) and a minority of whom are with negative or unknown evidence of autoantibodies (92–98).

There were some similarities among these patients in the aspect of clinical features, despite of their different MSAs and myositis-associated autoantibodies (MAAs). Firstly, SIH is a short-term complication of MDA5^+^ DM, usually occurring within 1-2 months after the onset of MDA5^+^ DM. However, the causes or incentives of SIH are unknown. SIH among all the reported patients appeared suddenly. They mainly presented focal muscle pain even with the complication of hypovolemia owing to the hemorrhage. Secondly, by means of CT and angiography, the responsible vessels could be found. The involved vessels were usually large blood arteries located in the iliolumbar regions, abdomen, shoulders and limbs. The involved muscles often lied in the deep muscle groups (e.g., iliopsoas and psoas). More than one blood vessel may be involved in an individual. Owing to the involvement of arteries, the bleeding progresses rapidly, resulting in further rebleeding after hemostasis, rapid decrease of hemoglobin, DIC or hemorrhagic shock (89, 99). Only one case showed skin bruising (93). It is supposed that SIH may exist in different DMs and is not specific to MDA5^+^ DM. But more reports and research are needed to support this.

Notably, muscular symptoms of MDA5^+^ DM may cover up the onset of SIH. Although MDA5^+^ DM is a form of CADMs and its myopathic features are usually absent, it sometimes presents mild muscular features. However, SIH may also present muscular features (including regional pain and swelling), which may mix with the existing muscular symptoms of MDA5^+^ DM. The clinicians may have difficulty in distinguishing between these two and even ignore SIH. Therefore, when patients feel myalgia and muscular swelling, imaging should be performed to identify SIH in DM patients.

### Diagnosis

The identification of SIH requires imaging and angiography. Through computed tomography (CT), hematoma could be seen surrounding the muscles. Angiography could be used to further determine the responsible vessels and even small aneurysms (94). In the meanwhile, it is also necessary to exclude some potential risk factors, such as dysfunction of hemostasis and coagulation, and the use of anti-coagulants or anti-plate drugs. Some cases showed prolonged prothrombin time (PT) and activated partial thromboplastin time (APTT). Some patients were inflicted on SIH after the prophylactic use of anticoagulant and anti-plate drugs. However, some cases had normality of partial results before or after the use of anticoagulant and anti-plate drugs, but still presented SIH. Therefore, the level of hemostasis and coagulation should be monitored regularly during the hospitalization and anti-platelet and anticoagulant drugs should be carefully used. A comparison of indexes before and after the presentation of SIH should be also needed.

### Pathogenesis

The pathogenesis of SIH in MDA5^+^ DM is ill-informed. It is likely related to the damage of vessels. Angiography can find hemorrhage in large and medium-sized vessels, but microvascular damage is difficult to perceive. It was reported that there are some biomarkers (such as endothelin and vWF) increased in MDA5^+^ DM patients with cutaneous ulcers, suggesting the correlation between MDA5^+^ DM and endothelial injury (32). A muscle biopsy of an anti-SSA/Ro-52-positive DM patient with SIH showed intravascular coagulation and capillary disruption (90). Another study on muscle pathology also showed capillary loss was apparent in non-MDA5^+^ DM patients, while interestingly tissue specimens showed reserved capillary density and infiltrated inflammation clustering focally in the perimysium around a single vessel in MDA5^+^ DM (100). Perhaps, SIH of MDA5^+^ DM is different from that of non-MDA5^+^ DM at the aspect of vessel involvement. It is hypothesized that SIH is associated with peripheral inflammation of a single vessel rather than the extensive destruction of capillaries around muscle tissues. Furthermore, SIH may be also related to the treatment of MDA5^+^ DM. Most MDA5^+^ DM patients are often treated with high-dose glucocorticoids as a priority. Glucocorticoids may influence protein synthesis and cause muscle consumption. The depleted muscle tissues lose their compression and vasoconstriction effect on the peripheral blood vessels when the vessels rupture. Together, SIH may be the result of vessel damage and drug use, but more attention is needed.

### Treatment

As for the treatment of SIH, hemostatic therapy, vascular embolization and bypass surgery are used and generally effective. When bleeding is controlled, patients may die from other complications of MDA5^+^ DM such as respiratory failure or infection instead of hemorrhage. Notably, when using high-dose glucocorticoids for MDA5^+^ DM, we should pay attention to its adverse effects of muscle consumption (**Figure 3**).

## Conclusion

In conclusion, a definitive diagnosis of MDA5^+^ DM should be established first before the diagnosis of complications. No matter what complications the patients are complicated with, it is necessary to pay attention to the occurrence and prevention of them as early as possible. Sometimes the primary symptoms of MDA5^+^ DM may be confused with those complications. Therefore, physicians should shed light on the relationship between MDA5^+^ DM and the rare complications. In the meanwhile, it is supposed for the physicians to make a diagnosis early and intervene in time to nip them in the bud. In the future, more research needs to be warranted and published.

## Author contributions

JY: writing and figures. BY: concept and proof reading. Both authors contributed to the article and approved the submitted version.

## Funding

This research was funded by grant from the National Natural Science Foundation of China (No. 81871285).

## Conflict of interest

The authors declare that the research was conducted in the absence of any commercial or financial relationships that could be construed as a potential conflict of interest.

## Publisher’s note

All claims expressed in this article are solely those of the authors and do not necessarily represent those of their affiliated organizations, or those of the publisher, the editors and the reviewers. Any product that may be evaluated in this article, or claim that may be made by its manufacturer, is not guaranteed or endorsed by the publisher.

## Abbreviations

A-aDO_2_, alveolar-arterial oxygen difference; AIP, acute interstitial pneumonia; APTT, activated partial thromboplastin time; ARDS, acute respiratory distress syndrome; AST, elevated aspartate transaminase; CADM, clinically amyopathic dermatomyositis; CCL2, C-C motif chemokine ligand 2; COVID-19, coronavirus disease 2019; CT, computed tomography; CTD, connective tissue disease; CXCL, C-X-C motif chemokine ligand; DAD, diffuse alveolar damage; DIC, disseminated intravascular coagulation; DM, dermatomyositis; HLH, hemophagocytic lymphohistiocytosis; HRCT, high-resolution computed tomography; IFN-I, type I interferon; IIM, idiopathic inflammatory myopathy; IL, interleukin; IL-1R1, IL-1 receptor type 1; IL-1RA, IL-1 receptor antagonist; IVIG, intravenous immune globulin; JAK, Janus kinase; JDM, juvenile dermatomyositis; LDH, lactate dehydrogenase; MAA, myositis-associated autoantibody; MAS, macrophage activation syndrome; MDA5, melanoma differentiation-associated gene 5; MRI, magnetic resonance imaging; MSA, myositis-specific autoantibody; NK, natural killer; NPPV, noninvasive positive pressure ventilator; PBMC, peripheral blood mononuclear cell; pDC, plasmacytoid dendritic cells; PET/CT, positron emission tomography/computed tomography; PNM, pneumomediastinum; PT, prothrombin time; RIG-I, retinoic acid-inducible gene I; RP-ILD, rapidly progressive interstitial lung disease; SIH, spontaneous intramuscular hemorrhage; STAT, signal transducer and activator of transcription; TYK, tyrosine kinase; TNF, tumor necrosis factor; vWF, von Willebrand factor.
